# Non-electrolytic synthesis of copper oxide/carbon nanocomposite by surface plasma in super-dehydrated ethanol

**DOI:** 10.1038/srep21178

**Published:** 2016-02-16

**Authors:** Dmytro S. Kozak, Ruslan A. Sergiienko, Etsuro Shibata, Atsushi Iizuka, Takashi Nakamura

**Affiliations:** 1 Institute of Multidisciplinary Research for Advanced Materials (IMRAM), Tohoku University, 1, 1 Katahira, 2-Chome, Aobaku, Sendai 980-8577, Japan; 2 Physico – Technological Institute of Metals and Alloys of National Academy of Science of Ukraine(PTIMA), Vernadsky Ave, 34/1, 03142 Kyiv, Ukraine

## Abstract

Electrolytic processes are widely used to synthesize different nanomaterials and it does not depend on what kind of the method has been applied (wet-chemistry, sonochemistry, plasma chemistry, electrolysis and so on). Generally, the reactions in the electrolyte are considered to be reduction/oxidation (REDOX) reactions between chemical reagents or the deposition of matter on the electrodes, in line with Faraday’s law. Due to the presence of electroconductive additives in any electrolyte, the polarization effect of polar molecules conducting an electrical current disappears, when external high-strength electric field is induced. Because initially of the charge transfer always belongs of electroconductive additive and it does not depend on applied voltage. The polarization of ethanol molecules has been applied to conduct an electric current by surface plasma interaction for the synthesis of a copper oxide/carbon nanocomposite material.

Many methods are available for synthesis of copper oxide (Cu_2_O/CuO) nanoparticles of various sizes, shapes and morphologies^1^,^2^,^3^,^4^. The physico-chemical properties of copper oxide nanoparticles allow these materials to be potential candidates for use in electrodes materials, solar energy conversion devices, sensors, antibacterial activity, and catalysts^5^,^6^,^7^,^8^,^9^,^10^,^11^. Some research groups^12^,^13^,^14^ have synthesised and demonstrated copper oxide/graphene nanocomposite an anode materials for lithium-ion battery. Yu-Wei Hsu and co-workers^15^ have studied the electrochemical detection of glucose using a copper oxide/graphene-modified glassy carbon electrode. High electrocatalytic activity for nitrite detection in aqueous media was reported in the copper oxide modified carbon powder – epoxy composite electrodes^16^, a copper oxide/carbon nanotube nanocomposite for the electrocatalytic reduction of nitrates was proposed^17^, and a modified nano-sized copper oxide/multi-wall carbon nanotubes/Nafion composite film–modified electrode was fabricated which showed a sensitive and selective determination of dopamine^18^. Generally, the preparation of the copper oxide/carbon nanocomposites includes electrolytic process synthesis of copper oxide and its mixture with the chosen carbon nanomaterials (such as graphene, graphene oxide, nanotubes and graphite) at the required volume^12^,^13^,^14^,^15^,^16^,^17^,^18^.

We developed a simple and novel method for copper oxide/carbon nanocomposite synthesis which includes only one fabrication step without additional mixture with carbon materials and any of electroconductive additives. In this respect, our method differs considerably from traditional electrolyte system methods. The new developed method includes two independent processes: 1) growth of carbon dendrites on the cathode surface above the super-dehydrated ethanol as described in ref. 19 and 2) the migration of copper atoms from the anode through the liquid organic phase to the area of surface plasma, where the copper oxide/carbon composite begins to be formed. This method bases on the polarization of the organic molecules, which conduct an electric current under the influence of external a high-strength electric field without the use of electroconductive additives; therefore, the applied electrochemical processes differs from Faraday’s process at the electrodes.

## Surface plasma generation

Recently, the surface plasma process undergone much research that indicated the possibilities for new uses in the future. The basic points of plasma liquid chemistry, including those of surface plasma above liquid are described in ref. 20, 21, 22; reference analysis allowed the conclusion that electroconductive additives were always added to the liquid phase. Thus, a significant contribution of the charge transfer capability of polar liquid belongs to the solvated ions of the electroconductive additives initially; the effect of molecules polarization disappears, when a external high-strength electric field is induced. In the synthesis presented here, we have excluded any electroconductiven additives in ethanol, so the surface plasma process can be described as follows. Ethanol is a polar liquid with a dielectric constant of ε = 25, meaning that the ethanol molecules have dipole moments even in the absence of an external electric field. The dipole moments of the ethanol molecules are maintained in both the liquid and gas phase. The dipoles are aligned with the direction of external electric field. Therefore, the electronic/dipole-relaxation polarization occurs in the liquid ethanol.

**Figure 1:**
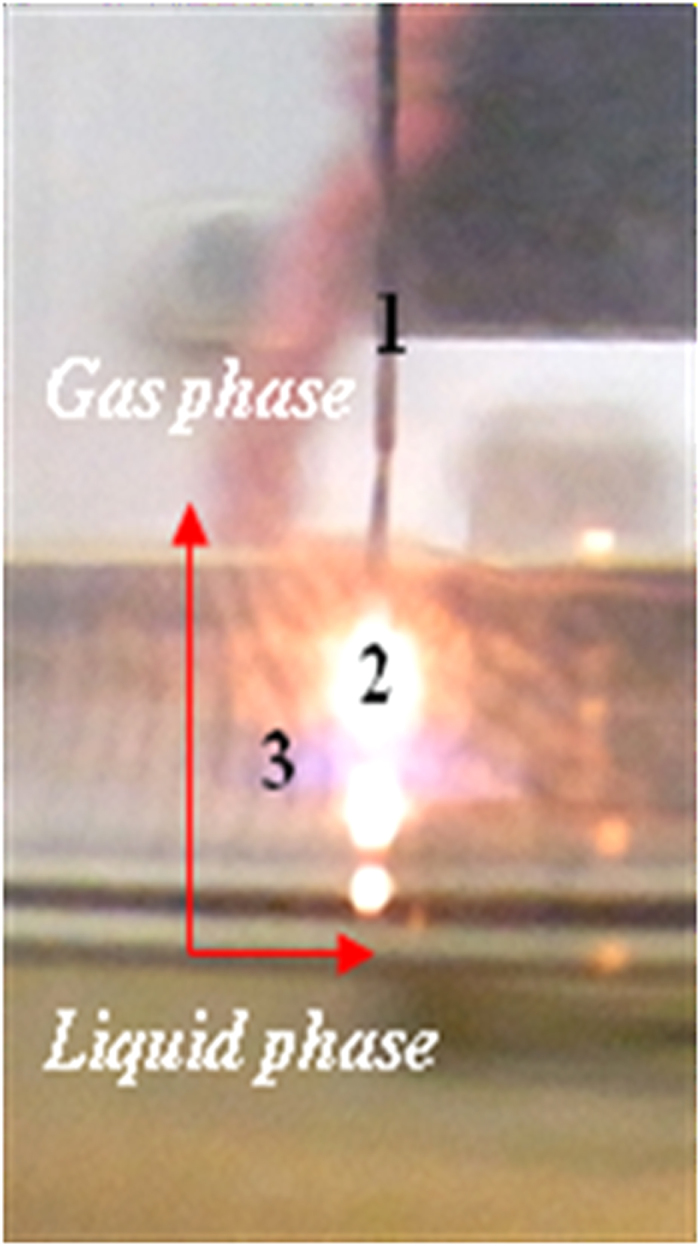
A photo-scheme of the surface plasma process. **(1)** Carbon dendrite, **(2)** hot-spot, and **(3)** the surface-liquid plasma area. The red arrows indicate the direction of gas phase formation and the boundary between the plasma and the liquid ethanol surface.

In the present experiment, the external electric field was non-uniform in strength. The density of the dipoles was maximized in the cathode region located above the liquid ethanol surface. The dipole moments of the ethanol molecules aligned with the external electric field in both the liquid ethanol phase and the gas phase mixture of argon with ethanol. The maximum electric field strength occurred below the cathode, and electronic breakdown was initiated between the cathode and the liquid ethanol surface, as shown in Fig. 1. The ionization of argon atoms and the decomposition of the ethanol molecules occurred as a result of the electronic breakdown, permitting the conduction of an electric current in the circuit.

## Formation of copper oxide/carbon nanocomposite

The process utilized in this work, can be similar to that used for the synthesis of an alkoxides by the anodic dissolution of various metals in anhydrous electrolyte alcohols. In both cases anodic oxidation occurred, but the initial mechanisms differed. The surface plasma process is classified as a non-equilibrium process under near-atmospheric pressure, in which the quantities of formed negative (including electrons) and positive charged species are not equal per unit time^20^,^21^,^22^. If electroconductive additives existed in the anhydrous electrolyte alcohols as a result of the formation of a stable insoluble complexes of metal alkoxide^22^ (in copper alkoxide, these complexes are colored), then anodic oxidation would occur after a certain time in the surface plasma process. The generation of surface plasma above the super-dehydrated ethanol is based on the polarization effect of the polar ethanol molecules under the influence of the external high-strength electric field, which allowed the conduction of an electric current. In this latter, case the formation of copper alkoxides could not occur, because the electrons initiated an electric current in the circuit. This process is complicated the convection, light irradiation induced by the surface plasma and parallel physicochemical reactions occurring simultaneously at the anode and the surface plasma region. The differences between the two reactions allowed the consideration of other possible physicochemical reaction mechanisms in different time intervals.

**Figure 2:**
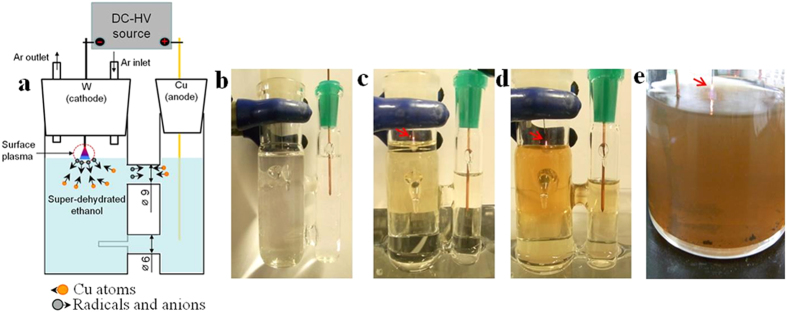
Experimental setup (**a**) and the surface plasma process in action for synthesis of copper oxide/carbon nanocomposite (**b**–**e**). (**a**) The migration of copper atoms and of radicals and anions moving in the initial time. (**b**) Colorless liquid in the cathode region before the experiment and brazed liquid after plasma burning for 3 (**c**); 10 (**d**) and 25 (**e**) minutes, respectively. Red arrows indicate the surface plasma process in action (**c**–**e**). Argon gas flow was fixed as protective atmosphere in our experiment.

The most dramatic difference between anodic oxidation in anhydrous electrolyte alcohols and the surface plasma process seemed to occur initially, when the migration of copper atoms was possible under the external high-strength electric field, and when an electrical current passes in the system circuit. As a copper atom migrated from the anode, the ethanol molecules decomposed to form radicals and anions during the surface plasma interaction; the generated radicals and anions moved to the anode direction under an external high-strength electric field, as demonstrated in Fig. 2a.

While the copper atoms were in motion under the external high-strength electric field, some were attacked by the radicals and anions to form copper oxide in liquid phase, but some of the copper oxides formed close to the surface plasma region initially, as shown in Fig. 2b,c. Some particles were transferred away by convection from the surface plasma region to become distributed in the liquid organic phase volume, as demonstrated on Fig. 2d. The decreasing volume of organic liquid in the right (anode) side of the reaction connected vessel was attributed partially to the behavior feature of dielectric liquid under an external induced high-strength electric field and partially to ethanol evaporation, but not with the formation of a gas phase at the anode (see Fig. 2b–d). This was because, when the external high-strength electric field was interrupted to induce of electric current in the circuit, the volume of organic liquid was restored after a certain time elapses.

While the process continued, the level of radicals and anions increased in the organic liquid volume; the surface of the copper anode was easily oxidized under these condition. In this period of time, the migration of the copper atoms was hindered and then the oxidation reaction at copper anode was directly occurred by reaction with radicals and anions, and the formation of the oxide/carbon nanocomposite continued (Fig. 2e).

It should be mentioned, when the copper electrodes were immersed in super-dehydrated ethanol and the external high-strength electric field was induced, copper atom did not migrate, because all electrical energy on the electrodes converted to heat which rapidly boiled the ethanol. This feature of the polar liquid was based on the dielectric loss. Thus, our experimental data confirmed that, in the case of copper migration from an anode in an ethanol liquid phase, the color of the organic solvent remained constant throughout the experiment regardless of the green Cu^+^ and blue Cu^2+^ ions (see Fig. 2e). The copper ethoxide (Cu(OC_2_H_5_)_2_) powder has colors varying from green the blue and is commercially available^23^.

## Observation of copper oxide/carbon nanocomposite

**Figure 3:**
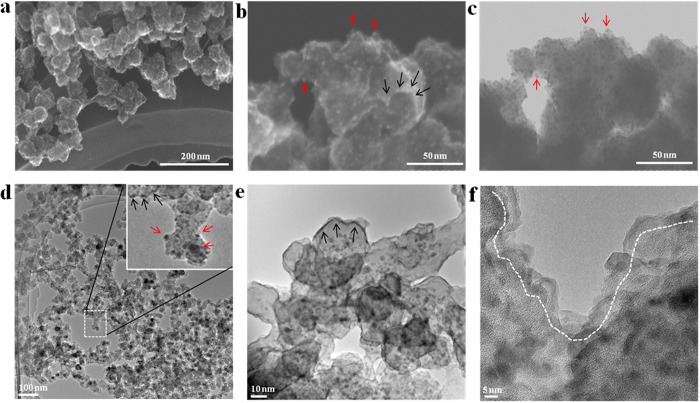
Copper oxide/carbon nanocomposite synthesized by surface plasma in super-dehydrated ethanol for 10 minutes. (**a**) General SEM image of copper oxide/carbon nanocomposite, (**b**) secondary electron emission (STEM) image of copper oxide particles in carbon matrixes and (**c**) BF-STEM image of the same place. (**d**) HRTEM image of copper oxide/carbon nanocomposite network, (**e**) the multiplicity of carbon leafs with apparent boundaries and (**f**) the multiple boundaries of the copper oxide/carbon nanocomposite.

Figure 3 shows the copper oxide/carbon nanocomposite on a molybdenum-mesh. Generally, the copper oxide/carbon nanocomposite can be described as a structure similar to beads. The majority of the beads are connected to the network (Fig. 3a). The scanning transmission electron microscope (STEM) image permits the observation of some copper oxide particles attached over the carbon surface (red arrows in Fig. 3b); the features of the carbon-beads matrix structure are clearly seen. The carbon-beads matrix consists of a multiple leaves of carbon (black arrows in Fig. 3b) with uniformly dispersed spherical copper oxide nanoparticles (Fig. 3c). A bright-field STEM image revealed the internal structure and distribution of the copper oxide in the nanocomposite; a majority of copper oxide particles are presented in the middle of the carbon-beads matrix (Fig. 3c).

High-resolution TEM observation of the carbon-beads matrix allowed the identification some features of the matrix’s network structure (Fig. 3d). The red and black arrows point to the copper oxide particles on the outer surface of the carbon-beads matrix and the apparent boundaries of carbon leaf, respectively (Fig. 3d,e). The boundaries of multiple carbon leaves are shown in Fig. 3f, above the dotted line, but the boundary itself has a clustered structure including both copper oxide nanoparticles and carbon.

**Figure 4:**
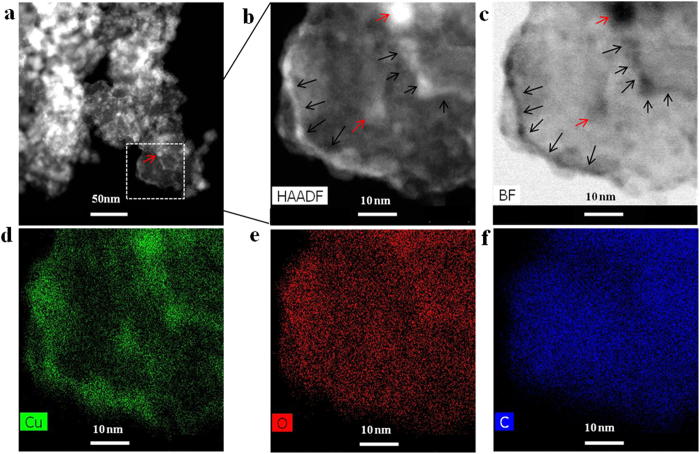
Element mapping of a selected carbon leaf in the copper oxide/carbon nanocomposite synthesized by surface plasma in super-dehydrated ethanol for 10 minutes. (**a**) HRTEM dark-field image of selected area of the copper oxide/carbon matrix, (**b**) dark- and (**c**) bright-fields HRTEM images of carbon-leaves with elemental distribution of (**d**) copper, (**e**) oxygen, and (**f**) carbon, respectively.

Element mapping analysis of a selected area demonstrated the enrichment of copper phase and the sizes of the copper oxide particles (Fig. 4a). In Fig. 4b,c, the copper oxide nanoparticles are shown in dark and brightness fields marked by red arrows; the particle size does not exceed 5 nm in diameter. The same images (Fig. 4b,c) present the multiplicity of carbon leaves enriched with the copper phase in the carbon-bead matrix; the apparent boundaries of the leaves are indicated by black arrows. Elemental copper is located along entire carbon leaf boundary, as demonstrated by the elongation of the enriched copper phase along carbon-leaf boundary (black arrows in Fig. 4b,c). The distribution of copper (Fig. 4d), oxygen (Fig. 4e), and carbon (Fig. 4f) in the selected area of the copper oxide/carbon nanocomposite was confirmed by energy-dispersive X-ray spectroscopy (EDX) analysis. We can assume that copper oxide clusters situate along entire periphery of carbon-leaf; in addition the spherical copper oxide nanoparticles distribute in the middle part of carbon leaf.

**Figure 5:**
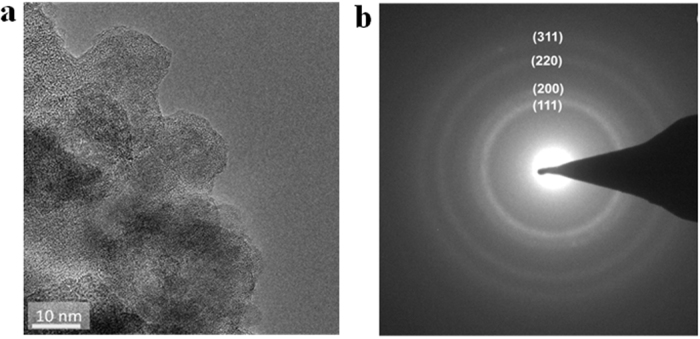
TEM image of copper oxide/carbon nanocomposite. (**b**) Corresponding SAED pattern of area presented in (**a**). Diffraction rings show the presence of univalent copper oxide (Cu_2_O) in the sample.

**Figure 6:**
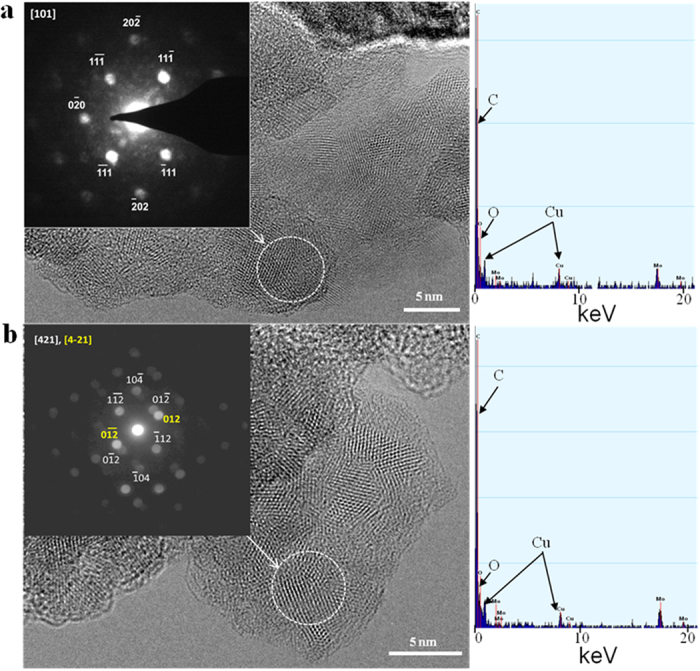
HRTEM images with corresponding nano-beam diffraction patterns and EDX data from selected locations on the copper oxide/carbon nanocomposite synthesized by surface plasma in super-dehydrated ethanol for 10 minutes. Select area electron diffraction (SAED) corresponding to various state oxidation of copper: (**a**) copper (I) oxide (Cu2O) and (**b**) copper (II) oxide (CuO). Energy-dispersive X-ray spectra shows molybdenum peaks from molybdenum mesh supporting the sample.

Despite on the complicated structure of the copper oxide/carbon nanocomposite, basic copper oxides phase of Cu_2_O and CuO could be identified. These copper oxide phases were identified by selected-area electron diffraction (SAED) and a nanobeam diffraction technique using an FEI Titan 80–300 Cubed TEM. The presence of cubic-phase Cu_2_O is confirmed by SAED from a larger sample area (Fig. 5a). The distinct diffraction rings in Fig. 5b have lattice spacing of 2.47 Å, 2.10 Å, 1.52 Å, and 1.28 Å indexed to the (111), (200), (220), and (311) reflections from univalent copper (I) oxide (Cu_2_O). We cannot exclude the presence of bivalent copper (II) oxide (CuO) in the synthesized copper oxide/carbon nanocomposite. Figure 6 shows the cluster structure of Cu_2_O and CuO phases on the periphery of the carbon-leaves. The interplanar spacings, as derived from the spots of the nanobeam diffraction pattern are 2.5 Å, 2.2 Å, and 1.5 Å, corresponding to the reference values of *d*_(111)_ = 2.46 Å, *d*_(200)_ = 2.46 Å, *d* = 2.25 Å and *d*_(220)_ = 1.51 Å, respectively, for the [101] zone-axis pattern of the cubic primitive lattice of Cu_2_O (PDF card #77–0199). Further observation of the cluster structure (Fig. 6a) of the copper oxide/carbon-leaves periphery identified a phase corresponding to CuO. The nanobeam diffraction pattern (insert in Fig. 6b) shows an array of diffraction spots consistent with lattice *d*-spacing of about 2.03 Å, 1.98 Å, and 1.29 Å. These *d*-spacings match the reference values of *d*_(012)_ = 2.05 Å, *d*_(11-2)_ = 1.96 Å, and *d*_(−104)_ = 1.272 Å for the different [421] and [4–21] zone-axes patterns of monoclinic CuO (PDF card #48–1548).

Thus, the HRTEM investigation revealed details of the internal structure of copper oxide/carbon-leaves and the structure of periphery carbon leaves. The periphery of the carbon leaves had a cluster structure including copper oxide in different oxidation states - copper (I) oxide (Cu_2_O) and copper (II) oxide (CuO). On basis of TEM investigations, we can assume that, when the external high-strength electric field was induced and the electric current was conducted in the circuit, copper atoms migrated through the liquid ethanol to the surface plasma region reaction, where copper oxide were formed.

It should be mentioned, that the carbon structure of the nanocomposite did not show crystallographic orientation, and HRTEM, electron beam diffraction and XRD investigations did not reveal the interplanar spacing of *d*_(002)_ = 3.4 Ǻ or X-ray/electron diffraction peaks from (002) glassy carbon (or pyrocarbon) atomic planes^19^.

## X-ray photoelectron and Raman spectroscopy analysis of copper oxide/carbon nanocomposite

**Figure 7:**
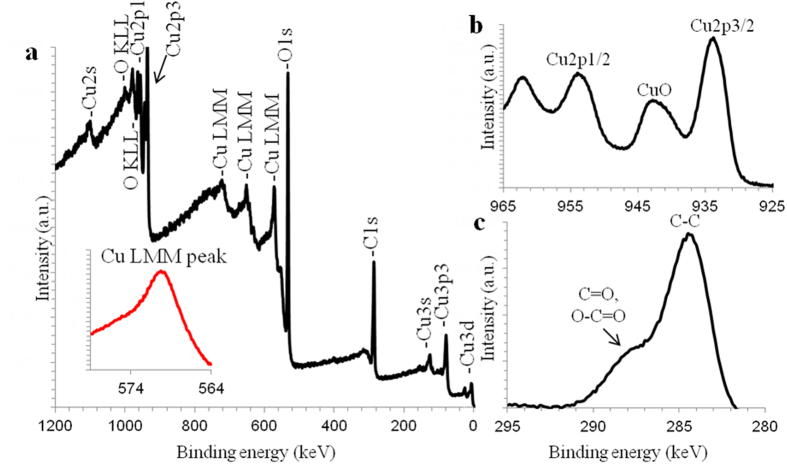
XPS spectrum of copper oxide/carbon nanocomposite synthesized by surface plasma in super-dehydrated ethanol for 10 minutes. (**a**) Wide spectra of copper oxide/carbon nanocomposite with insed of the Cu LMM peak shape to the Auger parameter, (**b**,**c**) high-resolution spectra of Cu2p3/2 and C1s, respectively.

The X-ray photoelectron spectroscopy analysis (XPS) data of the copper oxide/carbon nanocomposite are presented in Fig. 7a. The binding energy of the Cu2p3/2 peaks lies as typical in the of range 932.2–932.8 eV and 933.5–934.0 eV for Cu_2_O and CuO, respectively^24^. The peak analysis of Cu2p3/2 in Fig. 7b showed that the copper oxide in nanocomposite is in the bivalent oxidation state to CuO. The binding energy of the Cu2p3/2 peaks in copper oxide/carbon nanocomposite is 933.605 eV (Fig. 7b). Biljana Šljukić and co-workers^16^ showed a binding energy of Cu2p3/2 at 933.8 eV in their fabricated copper oxide/graphite composite. These data both correlate with the XPS database in ref. 24. The peak intensity of the Cu2p shake-up peak (marked as CuO in Fig. 7b) obtained for the copper oxide/carbon nanocomposite is close to the ratio characteristics of reference copper (II) oxides^16^,^24^.

The C1s peak has the highest intensity, demonstrating the presence of carbon in in the nanocomposite’s structure (Fig. 7a). The main peak at 284.593 eV corresponds to C-C bonding (Fig. 7c). The shoulders at about 288 eV and 287.70 eV correspond to C=O and O-C=O bonding energies, respectively^24^. The bonding energy for the chemical states of C-OH and C-O-C typically lies at 286.30 eV^24^. This suggests that the copper oxide has catalytic abilities in ethanol during the surface plasma process.

In additional to the Auger parameters of Cu2p3/2 peak position and shape, we used copper’s LMM peak and shape determine the chemical states of copper in our nanocomposite (inset in Fig. 7a). It is interesting that the main copper LMM peak at 569.986 eV corresponds to the copper peak position for univalent oxidation state; the binding energy for the bivalent copper oxidation state has a value of 568.25 eV^24^. The shape of the Cu LMM peaks for the copper oxidation states of Cu_2_O and CuO of the synthesized composite coincide with the shapes of reference Auger lines of Cu LMM^24^ in copper oxide compounds.

**Figure 8:**
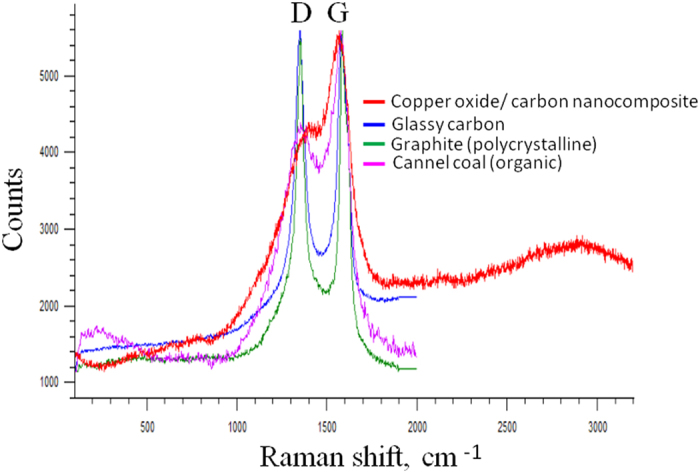
The Raman spectrum of copper oxide/carbon nanocomposite synthesized in super-dehydrated ethanol by surface plasma.

Raman-spectrum of the synthesized nanocomposite is shown in the Fig. 8. The carbon structure of copper oxide/carbon nanocomposite may correspond to reference polymeric amorphous carbon (a-C:H)^25^. Presented Raman data base of carbon structure closely relates to cannel coal (organic) (see Fig. 8) and we cannot exclude that synthesized carbon structure may correspond to desorbed carbon.

In summary, we discovered a new fabrication route for copper oxide/carbon nanocomposite in the area of non-equilibrium physicochemical reactions. The surface plasma interactions above super-dehydrated ethanol which is based on the polarization effect of polar organic molecules conduct an electrical current only under an external high-strength electric field. This process is advantageous in using only super-dehydrated ethanol, because the active radicals and anions are formed from the destruction of ethanol molecules by surface plasma after a certain time, while preventing copper ethoxide formation.

## Methods

### Cell preparation

The standard cell, usually applied for test of lithium-ion batteries, was used in our experiment as shown Fig. 2. One electrode was prepared from a 0.3-mm-diameter tungsten wire (99.9% purity, Nilaco), used as the cathode. The 3-mm-diameter copper (99.9% purity, Nilaco) anode of was directly immersed in liquid ethanol (Super Dehydrated (99.5%), water content 0.0005%, Wako). The copper anode was polished, rinsed by ethanol, and placed in glove-box before the experiment. The cell was assembled in the glove-box. The cathode was placed 3–5 mm above the ethanol surface. Protective argon gas was added to the cell at the rate 0.15 L/min.

### Electric parameters of surface plasma genaration

**Figure 9:**
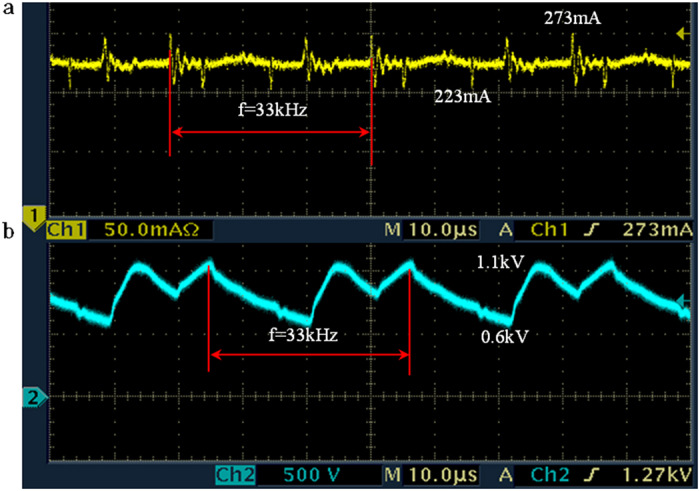
The oscillograph recording of the electric characteristics of surface plasma: (**a**) current and (**b**) voltage, respectively.

The surface plasma was generated using a high-voltage DC power supply with a pulsating frequency about 33 kHz. Figure 9 shows the voltage (V) and current (I) applied during the surface plasma process. The voltage and current show the pulsations about 33 kHz without a change in polarity. The values are I = 220 − 273 mA and V = 0.6 − 1.1 kV, as shown in Fig. 9a,b respectively.

At the beginning of the surface plasma process, the liquid ethanol temperature was approximately 20 °C. When the hot-spot was formed during the carbon dendrite growth, the temperature did not exceed 55–60 °C^19^.

### Copper oxide/carbon nanocomposite characterization

The structure and morphology of the as-prepared copper oxide/carbon nanocomposite was observed by scanning transmission electron microscope (STEM, Hitachi S5500). We employed a transmission electron microspore (TEM, Jeol JEM 3010EX and FEI Titan 80–300 Cubed TEM) operating at 300 kV to characterize the internal structure of the copper oxide/carbon nanocomposite.

### X-ray photoelectron and Raman spectroscopy analysis

The binding energies of the copper oxide/carbon in the nanocomposite were determined by X-ray photoelectron spectroscopy (XPS, ULVAC-PHI, Inc. PHI5600). Al K radiation (h*v* = 1486.6 eV) was employed for the photoelectron excitation. The preparation of the sample for XPS analysis was carried out in the glove-box. The sample was dried in the glove-box before studying, and after drying it was placed in a special container for XPS measurement.

The structure of copper oxide/carbon nanocomposite was characterized using a Ranishaw’s inVia Reflex Raman microscope.

## Additional Information

**How to cite this article**: Kozak, D. S. *et al.* Non-electrolytic synthesis of copper oxide/carbon nanocomposite by surface plasma in super-dehydrated ethanol. *Sci. Rep.* **6**, 21178; doi: 10.1038/srep21178 (2016).
